# Surgical outcomes in adults with purpura fulminans: a systematic review and patient-level meta-synthesis

**DOI:** 10.1186/s41038-019-0168-x

**Published:** 2019-10-18

**Authors:** Kevin M. Klifto, Caresse F. Gurno, Michael J. Grzelak, Stella M. Seal, Mohammed Asif, C. Scott Hultman, Julie A. Caffrey

**Affiliations:** 1 Department of Plastic and Reconstructive Surgery, The Johns Hopkins University School of Medicine, Baltimore, MD USA; 2 The Johns Hopkins University School of Nursing, Baltimore, MD USA; 3 Welch Medical Library, The Johns Hopkins University School of Medicine, Baltimore, MD USA; 4 Johns Hopkins Burn Center, 4940 Eastern Avenue, Baltimore, MD 21224 USA

**Keywords:** Amputation, Burn units, Neisseria, Purpura fulminans, Reconstructive surgical procedures, Shock, Septic, Surgeons, Treatment outcome

## Abstract

**Background:**

Cutaneous manifestations of purpura fulminans (PF) present many challenges for clinicians and surgeons. In a state of septic shock complicated by limb ischemia, surgical interventions are necessary to control the pathological cascade and improve patient outcomes. The objective of this article was to report etiologies and surgical outcomes associated with cutaneous manifestations in adults.

**Methods:**

This systematic review and meta-analysis compared 190 adult patients with etiologies, signs and symptoms, and surgical outcomes associated with cutaneous manifestations of PF. The PubMed, EMBASE, Cochrane Library, Web of Science, and Scopus databases were systematically and independently searched. Patient and clinical characteristics, surgical interventions, outcomes, and complications were recorded.

**Results:**

Seventy-nine studies were eligible for the systematic review, and 77 were eligible for meta-analysis using Preferred Reporting Items for Systematic Reviews and Meta-analysis (PRISMA) and Cochrane guidelines. A total of 71/190 (38%) cases reported surgical debridement. Fasciotomies were reported in 12/190 (6%) cases and 20 procedures. Amputations were reported in 154/190 (81%) cases. Reconstruction was reported in 45 cases. Skin grafts were applied in 31 cases. Flaps were used for reconstruction in 28 cases. Median (IQR) surgical procedures per patient were 4 (4, 5) procedures. Infectious organisms causing PF were 32% *Neisseria meningitidis* (*n* = 55) and 32% *Streptococcus pneumonia* (*n* = 55). Coagulase-negative *Staphylococcus* (95% confidence interval (CI)(8.2–177.9), *p* = 0.032), *Haemophilus influenza* (95%CI (7.2–133), *p* = 0.029), *Streptococcus pneumonia* (95% CI (13.3–75.9), *p* = 0.006), and West Nile Virus (95%CI (8.2–177.9), *p* = 0.032) were associated with significantly more extensive amputations compared to other organisms.

**Conclusion:**

This systematic review and patient-level meta-analysis found the most common presentation of PF was septic shock from an infectious organism. *Neisseria meningitidis* and *Streptococcus pneumonia* were equally the most common organisms associated with PF. The majority of cases were not treated in a burn center. The most common surgeries were amputations, with below-the-knee-amputations being the most common procedure. Skin grafting was the most commonly performed reconstructive procedure. The most common complications were secondary infections. Organisms with significantly more extensive amputations were coagulase-negative *Staphylococcus*, *Haemophilus influenza*, *Streptococcus pneumonia*, and West Nile Virus. Interpretation of findings should be cautioned due to limited sample data.

**Electronic supplementary material:**

The online version of this article (10.1186/s41038-019-0168-x) contains supplementary material, which is available to authorized users.

## Background

Cutaneous manifestations of purpura fulminans (PF) present many challenges for clinicians and surgeons [1]. The characteristic pattern of cutaneous purpura results from a pathological combination of disseminated intravascular coagulation (DIC) and endovascular thrombosis [2]. Activation of procoagulant and dysfunction of anticoagulant pathways with endothelial damage results in a systemic thrombotic state [2, 3].

Although first described in 1884 by Guelliot in children suffering from bacterial and viral infections, most children who developed PF have inherited deficiencies in protein C [2, 4]. This rare disorder presents in adults most commonly as an acute complication due to an abnormal response to systemic infections [5, 6]. *Neisseria meningitis* and *Streptococcus pneumonia* septicemia have been reported as an etiology in as many as 25% of children; however, data for adults is unknown [2, 7]. As PF progresses, secondary sequelae from hemorrhagic thrombotic infarcts and coagulopathy contribute to hemodynamic instability and subsequent limb ischemia, limb loss, morbidity, and mortality. In a state of septic shock complicated by limb ischemia, surgical interventions are necessary to control the pathological cascade and improve patient outcomes [1, 8].

This review provides healthcare providers with insight on outcomes and complications in adults presenting with PF. Patients and families can be informed early of realistic expectations and the need for surgical management when consenting to procedures.

We also report etiologies and surgical outcomes associated with cutaneous manifestations in adults. This article is an attempt to organize the literature to create a uniform set of data for clinical interpretation and management to optimize outcomes and minimize complications.

## Methods

### Materials and methods

Preferred Reporting Items for Systematic Reviews and Meta-analysis (PRISMA) guidelines were followed throughout the literature search process to structure the framework for the review [9]. The structure for our systematic review is a similar format to prior reviews by members of our team.

#### Selection criteria

The participants, interventions, comparisons, outcomes, and study design (PICOS) strategy was followed for inclusion throughout the selection process. Participants were age ≥ 15 years for each study, treated as either inpatients or outpatients, diagnosed with PF (tissue biopsy or case reported), and had a surgical intervention. Interventions were surgical defined as debridement, amputation, or reconstruction (acellular dermal matrix (ADM), allografts, autografts, split-thickness graft, full-thickness graft, skin substitute, cutaneous flap, fascial flap, muscle flap, osseous flap, omental flap) and non-surgical (negative pressure wound therapy, hyperbaric oxygen) intended for direct management of PF. Comparisons were made between the different organisms or etiologies contributing to PF. Outcomes measured were PF etiologies, infectious organisms, burn center management, number of limbs involved, areas of necrosis, percent of total body surface area (%TBSA) involved, debridement (surgical excision), time to debridement in days, amputation, number of bones removed or transected at amputation, time to amputation in days, flaps, type of flap, time to flap in days, flap survival, skin graft, time to skin graft in days, type of skin graft, skin graft location, skin graft survival, protein C (drotrecogin alfa), intravenous immunoglobulin, antithrombin, plasmapheresis, negative pressure wound therapy, hyperbaric oxygen therapy, hospital length of stay (LOS) in days, total surgical procedures, significant improvement in PF reported following surgery, long-term patient follow-up in days, mortality, and time to mortality in days. Complications measured were scarring, wound contracture, thrombosis, progression of disease, wound infections or abscess, systemic infections, pressure ulcers, fistulas, and non-healing wounds. All outcomes and complications were reported as measured by studies. Study designs considered were randomized controlled trials (RCTs), clustered RCTs, non-randomized controlled trials, retrospective studies, observational studies, prospective studies, case-control studies, cohort studies, case series, and case reports. No predetermined length of follow-up for participants or specific years was considered for publication status. Studies were excluded if they were not in English, were reviews with no contribution of new data, non-peer reviewed literature, cadaver studies, animal studies, full-article were unavailable, patient death occurred prior to surgical management, studies not related to outcomes or complications discussed, and surgical interventions not performed for cutaneous manifestations of PF.

#### Search

A medical library informationist (SMS) conducted the initial literature search using five databases (MEDLINE via PubMed, EMBASE, Cochrane Library, Web of Science, and Scopus) from inception to April 4, 2019 (see Additional file 1). Reference lists of relevant articles were hand-searched to identify additional relevant studies. All references were imported into Covidence (Veritas Health Innovation Ltd., Melbourne, Australia) and reference management software. Duplicates were removed.

#### Data extraction

Two reviewers (KMK and CFG) systematically and independently performed the title/abstract screening, followed by a full-text review to ensure quality and accuracy throughout the process. Any disagreements regarding studies included or excluded were resolved by discussion. If disagreements were still present after discussion, a third reviewer (JAC) resolved remaining conflict. The following data were extracted qualitatively and quantitatively for outcome and complication variables of interest: authors, year of publication, type of study, sample size, male and female distributions, age, PF etiologies, infectious organisms, burn center management, number of limbs involved, areas of necrosis, %TBSA, debridement, time to debridement, amputation, time to amputation, flaps, type of flap, flap survival, skin graft, time to skin graft, type of skin graft, skin graft location, skin graft survival, protein C, intravenous immunoglobulin, antithrombin, plasmapheresis, negative pressure wound therapy, hyperbaric oxygen therapy, hospital LOS, total surgical procedures, significant improvement in PF reported following surgery, long-term patient follow-up, mortality and time to mortality. Complications measured were scarring, wound contracture, thrombosis, progression of disease, wound infections or abscess, systemic infections, pressure ulcers, fistulas, and non-healing wounds. If there were multiple reports from the same study, one data collection form was completed for the study from all of the reports to avoid duplicating results.

#### Quality assessment and unit of analysis issues

Two reviewers (KMK and CFG) assessed the risk of bias and study quality individually for each study at a patient data level. Study quality was assessed using the methodological quality and synthesis of case series and case reports [10].

Amputations were qualified as reported by each individual patient and quantified by the total surgical removal or transection bones of fingers and toes (1st digit = 2, 2nd–5th digits = 3), below-the-knee amputation (BKA = 28), above-the-knee amputation (AKA = 30), distal forearm (*n* = 29), and transhumoral (*n* = 30). Case reports that reported transtibial amputations, BKAs, and distal lower extremity amputations were all considered BKA. This was applied to other anatomical locations of the body. Total surgical procedures performed were tabulated only if the case report explicitly quantified the number of procedures. Time to debridement, time to amputation, time to the skin graft, hospital LOS, long-term patient follow-up, and time to mortality were converted to days if intervals were reported in hours, weeks, months, or years.

#### Data synthesis and statistical analysis

Descriptive statistics were applied to quantify male and female distributions, age, PF etiologies, infectious organisms, burn center management, number of limbs involved, areas of necrosis, %TBSA, debridement, time to debridement, amputation, time to amputation, flaps, type of flap, flap survival, skin graft, time to skin graft, type of skin graft, skin graft location, skin graft survival, protein C, intravenous immunoglobulin, antithrombin, plasmapheresis, negative pressure wound therapy, hyperbaric oxygen therapy, hospital LOS, total surgical procedures, significant improvement in PF reported following surgery, long-term patient follow-up, mortality, time to mortality, scarring, wound contracture, thrombosis, progression of disease, wound infections or abscess, systemic infections, pressure ulcers, fistulas, and non-healing wounds. We reported medians, interquartile ranges (IQR), and ranges for non-parametrically distributed data and means and standard deviations for parametrically distributed data. Meta-analysis was performed using individual patient data [11]. Outcomes associated with the different organisms were compared using logistic regression and Fisher’s exact test for dichotomous variables (amputation, complications, mortality) or linear regression and Mann-Whitney *U* test for continuous variables (bones removed or transected at amputation, time to amputation, hospital LOS) for a non-parametric distribution of data. All meta-analysis outcomes were two-tailed, with a significance level set at *α* of 0.05. Dichotomous and continuous outcomes were reported with 95% confidence intervals (CIs), *p* values, odds ratios (OR), and unstandardized beta coefficients. All meta-analyses were performed with IBM SPSS Version 25.0 (IBM Corporation, Redmond, Washington). No additional analyses were performed during our study.

## Results

### Study selection and characteristics

**Fig. 1:**
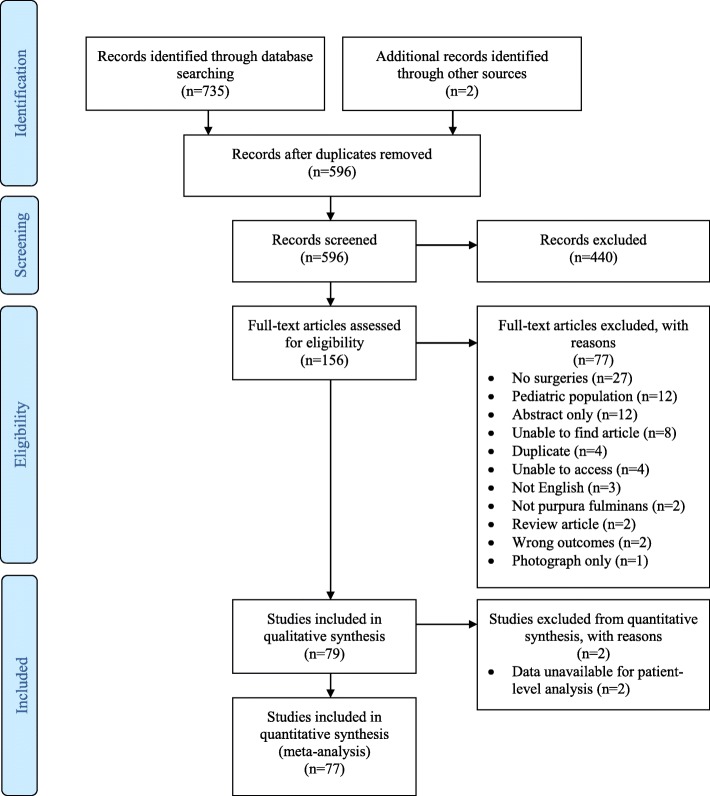
Preferred Reporting Items for Systematic Reviews and Meta-analysis (PRISMA) flow chart summarizes the results of the screening process and final article selections

The search resulted in 735 citations; after removing 141 duplicates, 594 citations remained. Following title/abstract review, 154 articles were eligible for full-text review. Manual review of references yielded two additional articles (*n* = 156). Following the full-text review, 79 articles were eligible for final data extraction and included in the systematic review (Fig. 1).

**Fig. 2:**
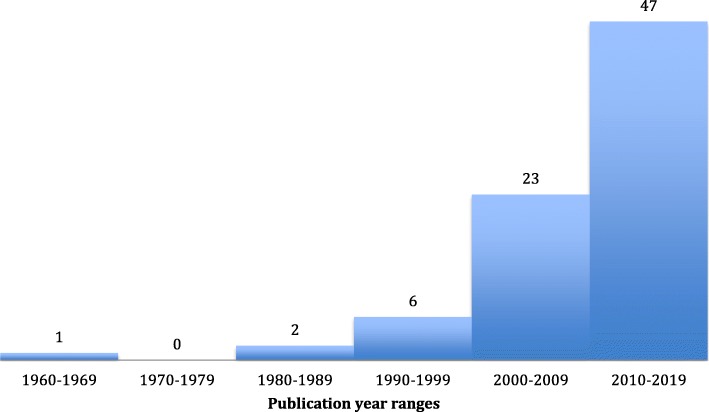
Number of purpura fulminans studies published per decade from 1968 to 2019

The 79 studies included in the systematic review were published from 1968 through 2019 (see Additional file 2: Table S1 and Additional file 3: Table S2) [1, 6, 8, 12–87]. The numbers of studies published per decade were one, 1960 to 1969; zero, 1970 to 1979; two, 1980 to 1989; six, 1990 to 1999; 23, 2000 to 2009; and 47, 2010 to 2019 (Fig. 2). A total of 190 patients age ≥ 15 years were diagnosed with PF and underwent a surgical procedure. There were 68 case studies [1, 6, 12–25, 27, 29, 31–40, 43–45, 47–61, 63, 65–72, 74, 75, 77–87], nine case series [8, 26, 30, 41, 42, 46, 64, 73, 76], one prospective cohort [62], and one retrospective cohort [28] studies published in English. All outcomes queried were identifiable during data extraction.

### Results and risk of bias of individual studies

Additional file 2: Table S1 and Additional file 3: Table S2 summarize the years, sample size, study design, age, sex, etiology, organism, necrosis, debridement, time to debridement, amputation, time to amputation, reconstruction, time to reconstruction, reconstruction location, hospital LOS, complications, mortality, and time to mortality, assessed by each individual study from the 79 articles included in our study. The quality of case-report and case-series studies included in the meta-analysis was provided for each study (see Additional file 4: Table S3).

### Synthesis of results across studies

Of the 79 studies included in the systematic review, 77 were included in the meta-analysis [1, 6, 8, 12–27, 29–61, 63–87]. Two studies were excluded due to inaccessibility of patient-level data to perform the quantitative synthesis. Of the outcomes queried, organism, amputations, time to amputation, burn center management, protein C, hospital LOS, complications, and mortality were evaluated by meta-analysis. Tables 1 and 2 summarize findings from qualitative and quantitative synthesis.

**Table 1:** Summary of findings for patient demographics from qualitative and quantitative synthesis

Total number of patients	Sex	Age, years Median (IQR)	Etiology	Infectious organism
190	• Males = 94 • Females = 89 • Unidentified = 7	40 (26–58)	• Infectious organism = 171 • Unknown = 9 • Not infectious = 6 • No organism found = 4	• *Neisseria meningitidis* = 55 • *Streptococccus pneumonia* = 55 • *Capnocytophaga canimorsus* = 9 • *Escherichia coli* = 7 • *Staphylococcus aureus* = 6 • *Streptococccus pyogenes* = 3 • *Klebsiella pneumonia* = 2 • *Haemophilus influenza* = 2 • *Plasmodium falciparum* = 2 • *Pseudomonas aeruginosa* = 1 • *Bacteroides fragilis* = 1 • *Capnocytophaga ochracea* = 1 • *Pasteurella multocida* = 1 • *Chlamydia pneumonia* = 1 • *Proteus mirabilis* = 1 • *Gemella bergeri* = 1 • *Vibrio vulnificus* = 1 • Leptospirosis = 1 • *Trichosporon asahii* = 1 • Cytomegalovirus = 1 • Varicella = 1 • West Nile Virus = 1

*IQR* interquartile range

**Table 2:** Summary of findings for patient outcomes from qualitative and quantitative synthesis

Total number of patients	Debridement	Time to debridement, days Median (IQR)	Amputations Median (IQR)	Amputations (bones removed/transected) Median (IQR)	Time to amputation, days Median (IQR)	Reconstruction (grafts)	Reconstruction (flaps)	Time to reconstruction, days Median (IQR)	Hospital LOS, days Median (IQR)	Complications	Mortality	Time to mortality, days Median (IQR)
190	71/190 cases	12.5 (1.8–20.3)	•154/190 cases •Limbs involved = 4 (4–4) •BKA = 64 •AKA = 30 •Below-the-elbow = 28 •Above-the-elbow = 3	56 (9–84)	18 (14–28)	•Total grafts = 33 •ADM = 3 •STSG = 13 •FTSG = 1 •Undifferentiated allografts = 5 •Undifferentiated autografts = 7 •Unknown = 4	•Total flaps = 28 •Latissimus dorsi = 7 •Orbicularis oris = 4 •Serratus anterior = 2 •Posterolateral thigh = 1 •Free ALT = 1 •Free medial gastrocnemius = 1 •Gracilis = 1 •Internal island saphenous = 1 •Free forearm = 1 •Lateral arm = 1 •Forehead = 1	39 (23–56)	57 (43.3–87.8)	•28/190 cases •Secondary infection = 20 •DVT/PE = 5 •Scarring = 5 •Wound contracture = 3 •Pressure ulcer = 1 •Wound breakdown = 1 •Alar stenosis = 1 •Facial deformity = 1 •Heterotopic ossification = 1	34/190 cases	36 (26.3–72.8)

*IQR* interquartile range, *BKA* below-the-knee amputation, *AKA* above-the-knee amputation, *ADM* acellular dermal matrix, *STSG* split-thickness skin graft, *FTSG* full-thickness skin graft, *ALT* anterolateral thigh, *LOS* length of stay, *DVT* deep vein thrombosis, *PE* pulmonary embolism

### Demographic

A total of 190 cases were identified through qualitative synthesis. Of these, 183 cases included 94 males and 89 females. Of the 190 cases included in the qualitative synthesis, 118 cases were included in the quantitative synthesis from 77 studies. Median (IQR) age was 40 years (26–58), ranging from 15 to 79 years. Median (IQR) limbs involved were four (4-4) limbs, and median (IQR) %TBSA was 45% (26–54%), ranging from 20–70%. Patients were treated in a burn center in 14/190 (7%) cases and eight studies.

### Etiology

The most common etiology reported for qualitative synthesis was an infectious organism (*n* = 171), followed by no organisms or unknown statuses of organisms reported in 19 patients. Infectious organisms causing PF were 32% *Neisseria meningitidis* (*n* = 55/171), 32% *Streptococcus pneumonia* (*n* = 55/171), 9% other organisms (*n* = 16/171), 5% *Capnocytophaga canimorsus* (*n* = 9/171), 4% *Escherichia coli* (*n* = 7/171), 4% *Staphylococcus aureus* (*n* = 6/171), *Streptococcus pyogenes* (*n* = 3), *Klebsiella pneumonia* (*n* = 2), *Haemophilus influenza* (*n* = 2), *Plasmodium falciparum* (*n* = 2), *Pseudomonas aeruginosa* (*n* = 1), *Bacteroides fragilis* (*n* = 1), *Capnocytophaga ochracea* (*n* = 1), *Pasteurella multocida* (*n* = 1), *Chlamydia pneumonia* (*n* = 1), *Proteus mirabilis* (*n* = 1), *Gemella bergeri* (*n* = 1), *Vibrio vulnificus* (*n* = 1), Leptospirosis (*n* = 1), *Trichosporon asahii* (*n* = 1), Cytomegalovirus (*n* = 1), Varicella (*n* = 1), and West Nile Virus (*n* = 1). No organisms or unknown statuses of organisms were reported in 19 patients. Identifiable non-infectious causes included macrophage activating syndrome, small cell lung cancer, breast necrosis, medication-induced fluoroquinolones, and postpartum. The most common etiology included in quantitative synthesis was an infectious organism (*n* = 99/118). The most common patient presentation was septic shock. Septic shock was reported from an infectious organism in 84/99 cases.

### Burn center management

Eight studies (14/190 cases) reported patient transfer to a burn center for higher level care. Median (IQR) days until patient transfer were 14 (10–15), ranging from 1 to 28 days after initial hospital admission. Amputations occurred in 11/14 patients transferred to a burn center compared to 70/104 patients not transferred to a burn center (*p* = 0.385). Median (IQR) extent of amputations were 61 (56–67) for patients transferred to a burn center compared to 56 (5.25–84) for patients not transferred to a burn center (*p* = 0.248). Median (IQR) hospital LOS was 78 (35–140) days for patients transferred to a burn center compared to 57 (44–85) days for patients not transferred to a burn center (*p* = 0.585). Six complications occurred in 14 patients transferred to a burn center compared to 23 complications in 104 patients not transferred to a burn center (*p* = 0.189). Mortality occurred in 3/14 patients transferred to a burn center compared to 11/104 patients not transferred to a burn center (*p* = 0.385). These findings were not significant.

### Non-surgical interventions

Protein C use was reported in 23/190 (12%) cases, antithrombin use in 5/190 (3%) cases, immunoglobulin use in 2/190 (1%) cases, and plasmapheresis use in 3/190 (2%) cases. Hyperbaric oxygen use was reported in 3/190 (2%) cases. Negative pressure wound therapy use was reported in 9/190 (5%) cases.

### Surgical interventions

A total of 71/190 (38%) cases reported surgical debridement. Median (IQR) time to debridement was 12.5 (1.8–20.3) days, ranging from one to 42 days. Fasciotomies were reported in 12/190 (6%) cases and 20 procedures. Amputations were reported in 154/190 (81%) cases. Median (IQR) time to amputation was 18 (14–28) days, ranging from one to 56 days. There were 64 BKAs, 30 AKAs, 28 below-the-elbow, and three above-the-elbow amputations. Reconstruction was reported in 45 cases. Median (IQR) time to reconstruction was 39 (23–56) days, ranging from 13 to 224 days. Skin grafts were applied in 33 cases. There were three ADMs, 13 split-thickness grafts, one full-thickness graft, five undifferentiated allografts, seven undifferentiated autografts cases, and four unknown grafts. Graft survival was reported in eight cases. Flaps were used for reconstruction in 28 cases. There were seven latissimus dorsi, two serratus anterior, four orbicularis oris, one posterolateral thigh, one free anterolateral thigh, one free medial gastrocnemius, one gracilis, one internal island saphenous, one free forearm, one lateral arm, and one forehead flap. Median (IQR) surgical procedures per patient were 4 (4, 5) procedures, ranging from two to eight procedures. Table 3 compares the extent of amputations, associated with different organisms. Coagulase-negative *Staphylococcus* (95%CI (8.2–177.9), *p* = 0.032), *Haemophilus influenza* (95%CI (7.2–133), *p* = 0.029), *Streptococcus pneumonia* (95%CI (13.3–75.9), *p* = 0.006), and West Nile Virus (95%CI (8.2–177.9), *p* = 0.032) were associated with significantly more extensive amputations compared to other organisms. There were no significant differences for times to amputation after comparing patients infected with the different organisms.

**Table 3:** Comparisons between the numbers of the bones removed or transected from patients during amputation with a specific organism using linear regression from quantitative data synthesis

Organisms	Number of the bones removed or transected, Median (IQR)	Unstandardized beta coefficient (bones removed or transected)	95%CI	*P* value
*Bacteroides fragilis*	28 (28–28)	1.152	[− 83.7–86]	0.978
*Capnocytophaga canimorsus*	54 (11–63)	21.425	[− 16.5–59.3]	0.264
*Capnocytophaga ochracea*	84 (84–84)	60.980	[− 23.8–145.8]	0.156
*Chlamydia pneumonia*	0 (0–0)	− 28.762	[− 113.6–56.1]	0.501
Coagulase-negative *Staphylococcus*	114 (114–114)	93.030	[8.2–177.9]	*0.032*
Cytomegalovirus	0 (0–0)	− 28.762	[− 113.6–56.1]	0.501
*Escherichia coli*	56 (25–56)	32.361	[− 12.5–77.2]	0.115
*Gemella bergeri*	56 (56–56)	31.066	[− 53.8–115.9]	0.468
*Haemophilus influenza*	93 (79–106)	70.088	[7.2–133]	*0.029*
*Klebsiella pneumonia*	0 (0–0)	− 12.728	[− 75.6–50.2]	0.688
Leptospirosis	3 (3–3)	− 25.557	[− 110.4–59.3]	0.550
*Neisseria meningitidis*	56 (18–61)	20.207	[− 10.4–50.8]	0.192
No organism	60 (30–87)	32.136	[− 16.2–80.5]	0.190
*Pasteurella multocida*	59 (59–59)	34.271	[− 50.6–119.1]	0.423
*Plasmodium falciparum*	71 (50–93)	47.104	[− 15.8–110]	0.140
*Proteus mirabilis*	0 (0–0)	− 28.762	[− 113.6–56.1]	0.501
*Staphylococcus aureus*	75 (42–99)	41.780	[− 6.6–90.1]	0.089
*Streptococccus pneumonia*	85 (41–95)	44.603	[13.3–75.9]	*0.006*
*Streptococccus pyogenes*	90 (45–105)	45.692	[− 7.9–99.3]	0.094
*Trichosporon asahii*	0 (0–0)	− 28.762	[− 113.6–56.1]	0.501
Unknown	0 (0–84)	14.600	[− 27.8–57]	0.495
Varicella	14 (14–14)	− 13.805	[− 98.6–71]	0.747
West Nile Virus	114 (114–114)	93.030	[8.2–177.9]	*0.032*

*IQR* interquartile range, *CI* confidence interval

### Hospital LOS

Median (IQR) hospital LOS reported in 50/190 cases was 57 (43.3–87.8) days, ranging from 11 to 292 days. There were no significant differences in hospital LOS after comparing patients infected with the different organisms.

### Complications

Complications occurred in 28/190 cases. Secondary infections were the most common complication in 20 cases, followed by DVT/PE in five cases, scarring in five cases, contracture in three cases, pressure ulcer in one case, wound breakdown in one case, alar stenosis in one case, facial deformity in one case, and heterotopic ossification in one case. There were no significant differences in complications after comparing patients infected with the different organisms.

### Mortality

Mortality was reported in 34/190 (18%) cases. Median (IQR) time to mortality was 36 (26.3–72.8) days, ranging from 3 to 84. Median (IQR) follow-up was 365 (365–730) days, ranging from 28 to 2884 days. There were no significant differences in mortality after comparing patients infected with the different organisms.

## Discussion

This was the first systematic review and meta-analysis that compared infectious etiologies, signs and symptoms, and surgical outcomes associated with cutaneous manifestations in adults with PF. Most patients presented with septic shock due to infectious etiology. Similar to children, the most common infectious etiologies were *Neisseria meningitidis* and *Streptococccus pneumonia* in adults. Early identification of these organisms may allow the transfer of these patients to a higher level of care sooner to optimize patient outcomes. Patients had a median involvement of four limbs, %TBSA ranged from 20% to 70%, and 7% of cases were managed in a burn center. Assessing the involvement of four limbs and greater than 20% of the skin could play an important role for diagnostic criteria. Burn center management provides multidisciplinary staff trained to utilize resources for burn and non-burn skin wounds to improve patient outcomes [88]. The most common surgeries performed were amputations, with BKAs being the most common procedure. Skin grafting was the most commonly performed reconstructive procedure. Coagulase-negative *Staphylococcus*, *Haemophilus influenza*, *Streptococccus pneumonia*, and West Nile Virus were associated with more extensive amputations. Although coagulase-negative *Staphylococcus*, *Haemophilus influenza*, and West Nile Virus were associated with more extensive amputations, the small sample sizes for each organism were likely to over-estimate the effects of these results. The most common complications were secondary infections. Median hospital LOS was 57 days, ranging from 11 to 292 days. The rate of mortality was 18% from the 190 adult cases. Previous literature describes mortality ranging from 20% to 60%, with inclusion of data from pediatric populations [2]. Lower mortality rates observed in our study may be due to publication bias. Case reports involving patient deaths may be less likely to result in publication and appear in the literature. This effect may artificially lower the reported mortality rates.

Conservative surgical management should be considered for patients with PF. Surgical procedures interrupt anticoagulant administration and disrupt the skin barrier, resulting in susceptibility to secondary infections. During the early stages of PF, it is difficult to differentiate between viable and nonviable tissue. Extensive surgical management may result in unnecessary tissue loss. Avoiding early amputation in favor of autoamputation when applicable can minimize tissue loss and patient morbidity [89]. Fasciotomy is recommended for limb threatening ischemia and compromised blood flow from tissue edema [1, 90]. Reconstructive procedures may be delayed until the underlying cause and pathophysiological state of PF is controlled [34].

Studies that include surgical management of PF are primarily retrospectively designed. They are poor quality with many limitations. The majority of studies consisted of case reports, limiting use of forest plots, funnel plots, heterogeneity tests, and risks of bias. Times to debridement, times to burn center transfer, amputations, and reconstruction vary from study to study with conflicting results. Study populations vary from rural settings to cities, around the world, managed on standard hospital floors, intensive care units, and burn units. Studies ranged from 1968 through 2019, a 51-year time period. Our search revealed major gaps in reported data within the literature. Many studies reported regions of the body amputated, making it difficult to determine the level of transection. Types of skin grafts were not specified in many studies, along with many donor and recipient sites. Few studies differentiated the depth, locations and %TBSA of necrosis and surgical debridement. Many studies did not identify how patients were diagnosed with PF or etiologies. Non-surgical interventions were we underpowered in our study, limiting the interpretation of protein C administration for PF. Burn center management was reported in 14 cases. Some patients were transferred to a burn center after 28 days of hospital management, limiting the interpretation of benefits from early advanced management. Comparative groups of organisms in the meta-analysis were unequal sizes with varying reports of outcomes, complications, and follow-up. Non-parametric tests were implemented; however, the small sample sizes may have resulted in misinterpretation of non-significant findings. Significant findings should be interpreted carefully, not to over-estimate the effects of our findings.

All of these limitations demonstrate the importance for performing this systematic review and meta-analysis. By conducting this never performed review, we identified existing and future areas of research that can contribute a wealth of knowledge to the literature for healthcare providers, the public, and patients. In addition, we compared all available published data for adult surgical management of PF.

## Conclusion

The most common presentation of PF was septic shock from an infectious organism. Similar to the pediatric population, *Neisseria meningitidis* and *Streptococccus pneumonia* were equally the most common organisms associated with PF in adults. The majority of cases were not treated in a burn center; however, given the extensive skin and soft tissue involvement, a burn center may be better equipped to manage these complicated patients. Interpretation of findings should be cautioned due to limited sample data.

## Additional files

Additional file 1 : Search strategies (DOCX 19 kb)

Additional file 2 : Table S1. Case reports with surgical outcomes of purpura fulminans (DOCX 165 kb)

Additional file 3 : Table S2. Cohort studies with surgical outcomes of purpura fulminans (DOCX 50 kb)

Additional file 4 : Table S3. Methodological quality and synthesis of case series and case reports system [10] (DOCX 131 kb)

## Data Availability

The data used and/or analyzed during the current study are accessible online.

## Abbreviations

%TBSA: Percent of total body surface area
ADM: Acellular dermal matrix
AKA: Above-the-knee amputation
ALT: Anterolateral thigh
BKA: Below-the-knee amputation
CI: Confidence intervals
DIC: Disseminated intravascular coagulation
DVT: Deep vein thrombosis
FTSG: Full-thickness skin graft
IQR: Interquartile range
LOS: Length of stay
OR: Odds ratios
PE: Pulmonary embolism
PF: Purpura fulminans
PICOS: Participants, interventions, comparisons, outcomes, and study design
PRISMA: Preferred Reporting Items for Systematic Reviews and Meta-analysis
RCT: Randomized controlled trial
STSG: Split-thickness skin graft
