# Supplementary material for: Surgical outcomes in adults with purpura fulminans: a systematic review and patient-level meta-synthesis
Source: Burns Trauma. 2019 Oct 18;7:30. doi: 10.1186/s41038-019-0168-x (PMC6798408; doi:10.1186/s41038-019-0168-x)
Supplement: Supplementary file 1 — : Search strategies (DOCX 19 kb) [file 41038_2019_168_MOESM1_ESM.docx]

**Additional file 1.** Search Strategies

# Citation Totals

735 Total Citations

594 Remaining after Duplicates Removed

# Search Strategies by Database

## MEDLINE via PubMed

("Purpura Fulminans"[Mesh] OR "Purpura, Schoenlein-Henoch"[Mesh] OR "purpura fulminans" OR "fulminating purpura")

AND

(surgical procedures, operative [mh] OR surgery [sh] OR specialties, surgical [mh] OR surg* OR operation* OR perative*)

AND

(adolescent [mh] OR adult [mh] OR adoloscen* [tw] OR teen* [tw] OR adult* [tw] OR older* [tw] OR elder* [tw] OR senior* [tw])

AND

eng [la]

Searched: 04/05/2019

Citations retrieved: 426

## EMBASE

| No. | Query | Results |
| --- | --- | --- |
| #5 | #1 AND #2 AND #3 AND [english]/lim | **203** |
| #4 | #1 AND #2 AND #3 | **225** |
| #3 | 'adolescent'/exp OR 'adult'/exp OR adolescen*:ti,ab OR teen*:ti,ab OR adult*:ti,ab OR older*:ti,ab OR elder*:ti,ab OR senior*:ti,ab | **9,208,239** |
| #2 | 'surgery'/exp OR 'surgeon'/exp OR surg*:ti,ab OR operative*:ti,ab OR operation*:ti,ab | **5,944,208** |
| #1 | 'fulminating purpura'/exp OR 'fulminating purpura' OR ((purpura NEAR/3 (fulminan* OR fulminating)):ti,ab) | **1,684** |

## Cochrane Library

| ID | Search | Hits |
| --- | --- | --- |
| #1 | MeSH descriptor: [Purpura Fulminans] explode all trees | 0 |
| #2 | MeSH descriptor: [Purpura, Schoenlein-Henoch] explode all trees | 43 |
| #3 | (purpura NEAR/3 (fulminan* OR fulminating)) | 23 |
| #4 | #1 OR #2 OR #3 | 65 |
| #5 | MeSH descriptor: [Specialties, Surgical] explode all trees | 1734 |
| #6 | MeSH descriptor: [Surgical Procedures, Operative] explode all trees | 110990 |
| #7 | surg* OR operative* OR operation* | 266052 |
| #8 | #5 OR #6 OR #7 | 301289 |
| #9 | #4 AND #8 | 9 |

Searched: 04/05/2019 17:08:08

2 Cochrane Database of Systematic Reviews

7 Cochrane Central Register of Controlled Trials

## Web of Science

**TOPIC:** ((purpura NEAR/3 (fulminan* OR fulminating))) *AND* **TOPIC:** ((surg* OR operative* OR operation* OR surgery OR surgical OR surgeon*)) *AND* **TOPIC:** (adolescen* OR teen* OR adult* OR older* OR elder* OR senior*)

**Timespan:** All years. **Indexes:** SCI-EXPANDED, SSCI, A&HCI, CPCI-S, CPCI-SSH, BKCI-S, BKCI-SSH, ESCI, CCR-EXPANDED, IC.

Searched: 04/05/2019

Citations retrieved: 21

## Scopus

(TITLE-ABS-KEY(( purpura W/3 fulminan*) OR (purpura W/3 fulminat*)) AND TITLE-ABS-KEY (surgery OR surgeon* OR surgical* OR surg* OR operative* OR operation*) AND TITLE-ABS-KEY(adolescen* OR teen* OR adult* OR older* OR elder* OR senior*))

Searched: 04/05/2019

Citations retrieved: 76
