# Supplementary material for: Surgical outcomes in adults with purpura fulminans: a systematic review and patient-level meta-synthesis
Source: Burns Trauma. 2019 Oct 18;7:30. doi: 10.1186/s41038-019-0168-x (PMC6798408; doi:10.1186/s41038-019-0168-x)
Supplement: Supplementary file 2 — : Table S1. Case reports with surgical outcomes of purpura fulminans (DOCX 165 kb) [file 41038_2019_168_MOESM2_ESM.docx]

Supplemental Table 1. Case-reports with surgical outcomes of purpura fulminans

| Author | Year | Age | Sex | Etiology | Infectious organism | Necrosis | Debridement | Time to debridement (days) | Amputation | Bones removed/transected | Time to amputation (days) | Reconstruction | Time to reconstruction (days) | Reconstruction location | Hospital LOS (days) | Complications | Mortality | Time to mortality (days) |
| --- | --- | --- | --- | --- | --- | --- | --- | --- | --- | --- | --- | --- | --- | --- | --- | --- | --- | --- |
| Agarwal (12) | 2010 | 15 | F | Meningitis(not vaccinated) | *Neisseria meningitidis*, A | Bilateral hands and feet | Yes |  | 5 toes | 14 | 14 |  |  |  |  |  |  |  |
| Ahmad (13) | 2013 | 36 | M | Meningitis | *Neisseria meningitidis* | Toes, heels, limbs, nose, ears, penis | Yes, sharp | 32 | Multiple toes | 18 | 32 |  |  |  | 64 |  |  |  |
| Ahmad (13) | 2013 | 23 | M | Septic shock | *Neisseria meningitidis* | Limbs, feet, heels | Yes, sharp | 33 | 6 toes | 18 |  | Split-thickness graft | 44 |  | 104 |  |  |  |
| Amara (15) | 2014 | 53 | F | Septic shock(gastrectomy) | *Escherichia coli*, group A | Lower limbs | Cutaneous and subcutaneous tissues | 20 | 0 | 0 |  | Skin graft | 140 |  |  |  |  |  |
| Andreasen (14) | 2000 | 32 | F | Postpartum(asplenic) | *Streptococccus pneumoniae* | Upper and lower limbs | Multiple, right hand fasciotomy | 1 |  | 97 |  | ADM, ultrathin epidermal grafts |  |  | 252 | Recurrent sepsis |  |  |
| Arevalo (16) | 1997 | 21 | M | Fever, rash | *Neisseria meningitidis*, C | Upper and lower limbs | Tangental excision |  | Bilateral lower limbs, right hand, left hand fingers | 62 | 1 | Internal saphenous island flap, autografts |  | Patella, upper and lower limbs | 43 | reamputation of right lower limb, infection of left stump, ventilator associated pneumonia |  |  |
| Arnaiz-Garcia (17) | 2017 | 78 | F | Meningitis | *Neisseria meningitidis* | Toes and fingers | Yes |  | Bilateral BKA, fingers 2-5 of right hand | 76 |  |  |  |  |  |  |  |  |
| Bendapudi (18) | 2018 | 39 | M | Dog bite | *Capnocytophaga canimorsus* |  | Yes |  | Bilateral transmetacarpal and transmetatarsal | 14 |  |  |  |  | 57 |  |  |  |
| Bhatti (19) | 2019 | 29 | F | Postpartum | Unknown |  | Yes |  | 4th, 5th distal fingers of the left hand, and toes 9, 10 of both feet | 114 |  | Skin graft |  |  |  | Multiple wound infections |  |  |
| Bischof (20) | 2014 | 53 | M | Dog bite | *Capnocytophaga canimorsus* | Bilateral hands, feet, face |  |  | Bilateral BKA, bilateral transradial | 106 |  |  |  |  |  | Invasive aspergillosis | Yes | 18 |
| Bollero (21) | 2010 | 28 | M | Meningitis | *Neisseria meningitidis* | Right hand and fingers, extensor surfaces of elbows, lower legs, bilateral feet/toes | Lower limbs, right hand, elbows | 12 | Bilateral ankles and wrists | 56 |  | Alloplastic glycerolized skin graft, autologous split-thickness graft | 31, 47 | Lower limbs, right hand, elbows | 140 | Skin breakdown, bone exposure |  |  |
| Borges (22) | 2014 | 52 | M | Septic shock(dog bite) | *Pasteurella multocida* | Bilateral lower limbs, right index finger, nose | Nose skin and cartilage | 9 | All toes, first and fifth bilateral metatarsals, bilateral BKA | 59 | 27 |  |  |  |  |  |  |  |
| Chasan (23) | 1992 | 37 | M | Septic shock | *Neisseria meningitidis* | Bilateral upper and lower limbs, buttocks | Down to fascia | 14 | Bilateral BKA, index finger | 67 | 9 | Allograft, autograft from scalp, back, abdomen sites | 17 |  | 35 | Staphylococcus epidermidis wound infections |  |  |
| Choi (24) | 2005 | 70 | F | Septic shock | *Vibrio vulnificus* | Bilateral upper and lower limbs | Bilateral lower limbs to muscles |  | Bilateral BKA, left thumb, index, middle, ring fingers |  | 17 | Split-thickness graft | 56 | Bilateral lower limbs |  |  |  |  |
| Christiansen (25) | 2012 | 59 | F | Septic Shock(dog bite) | *Capnocytophaga canimorsus* | Left hand |  |  | 4th finger of left hand | 3 |  |  |  |  | 84 |  |  |  |
| Christiansen (25) | 2012 | 59 | M | Septic shock(dog licking wound) | *Capnocytophaga canimorsus* | Face, abdomen, bilateral upper and lower limbs | Bilateral lower limbs |  | Toes and both feet | 52 |  |  |  |  | 84 |  |  |  |
| Chu (26) | 1982 | 22 |  | Postsplenectomy | *Streptococccus pneumoniae* |  |  |  | 0 | 0 |  |  |  |  |  |  |  |  |
| Chu (26) | 1982 | 33 | F | Meningitis(not vaccinated) | *Neisseria meningitidis*, B | Bilateral upper and lower limbs, face, trunk | Yes |  | Bilateral AKA, unilateral forearm | 89 |  |  |  |  | 56 |  |  |  |
| Chu (26) | 1982 | 36 |  | Meningitis(not vaccinated) | *Neisseria meningitidis* |  | Skin on lower back |  | Bilateral BKA | 56 |  |  |  |  |  |  | Yes |  |
| Chu (26) | 1982 | 58 |  |  | Unknown |  | Yes |  | Bilateral BKA, fingers | 84 |  |  |  |  |  | Wound contracture |  |  |
| Chu (26) | 1982 | 60 | M |  | *Proteus mirabilis* | Toes |  |  | 0 | 0 |  |  |  |  |  |  | Yes | 16 |
| Cone (27) | 1982 | 60 | F | Septic shock(PE/DVT) | *Streptococccus pneumoniae*, serotype 16 | Lower limbs | Bilateral lower limbs | 4 | 0 | 0 |  |  |  |  |  |  |  |  |
| Dautzenberg (29) | 2017 | 56 | F | Septic shock | *Streptococccus pneumoniae* | Bilateral upper and lower limbs, face, trunk | Left leg, abdomen | 14 | Right lower limb | 28 | 14 |  |  |  |  | Nosocimial infection Candida albicans, Enterococcus faecium, Aspergillus fumigatus | Yes | 29 |
| Davis (30) | 2007 | 46 | M | Septic shock(asplenic) | *Streptococccus pneumoniae* | Bilateral upper and lower limbs |  |  | Bilateral BKA, unilateral below elbow | 85 |  |  |  |  |  |  |  |  |
| Davis (30) | 2007 | 77 | F | Septic shock | *Streptococccus pneumoniae* | Bilateral upper and lower limbs |  |  | Right foot, left foot transmetatarsal, right finger, distal index finger | 49 |  |  |  |  |  |  |  |  |
| Davis (30) | 2007 | 36 | F | Septic shock(dog bite) | *Capnocytophaga canimorsus* | Bilateral upper and lower limbs, tip of nose |  |  | Bilateral BKA | 56 |  |  |  |  |  |  |  |  |
| Davis (30) | 2007 | 56 | F | Small cell lung cancer |  | Bilateral upper and lower limbs |  |  | Multiple digits, partial right foot | 33 |  |  |  |  |  |  |  |  |
| Davis (30) | 2007 | 51 | M | Septic shock(pneumonia) | *Staphylococcus aureus* | Bilateral upper and lower limbs |  |  | Bilateral BKA, bilateral below elbow | 114 |  |  |  |  |  |  |  |  |
| De Salvia (31) | 2008 | 27 | M | Septic shock(asplenic) | *Streptococccus pneumoniae* | Nose | Nose |  |  |  |  |  | 56 | Nose |  | Recurrent fistula |  |  |
| Dedy (32) | 2016 | 68 | F | Septic shock(dog bite) | *Capnocytophaga canimorsus* | Finger tips, all toes | Right hand | <1 | Right index finger | 3 | 21 | Split-thickness graft | 21 | Right hand | 42 |  |  |  |
| Desai (33) | 2007 | 49 | M | Dental extraction, sepsis | *Capnocytophaga ochracea* | Ears, feet, hands trunk |  |  | Bilateral BKA, fingers | 84 | 56 |  |  |  | 87 |  |  |  |
| Duteille (34) | 2006 | 22 | M |  |  | Bilateral feet |  |  | All toes | 28 |  | 2 serratus anterior and latissimus dorsi flaps, | Right 70, left 84 |  | 84 |  |  |  |
| Duteille (34) | 2006 | 34 | M |  |  | Bilateral feet |  |  | All toes | 28 |  | 2 latissiums dorsi | Right 42, left 21 |  | 84 |  |  |  |
| Dykstra (35) | 2014 | 19 | F | Meningitis | *Neisseria meningitidis* | Bilateral upper and lower limbs, trunk | Bilateral forearm fasciotomies | 2 | Toes | 28 |  |  |  | Feet |  |  |  |  |
| El-Agwany (36) | 2017 | 27 | F | Hemorrhagic shock(postpartum) | Coagulase negative Staphylococcus | Bilateral upper and lower limbs | Right forearm fasciotomy | 10 | Bilateral BKA, bilateral below elbow | 114 |  |  |  |  | 28 |  |  |  |
| Endo (37) | 2014 | 41 | M | Septic shock(pneumonia) | *Haemophilus influenzae* | Bilateral upper and lower limbs |  |  | Bilateral AKA, right forearm, left upper arm | 119 | 9 |  |  |  |  | Methicillin-resistant Staphylococcus aureus sepsis | Yes | 34 |
| Eng (38) | 2014 | 66 | M | Septic shock(dog bite) | *Capnocytophaga canimorsus* | Nose, lower limbs, fingertips | Yes |  | Bilateral BKA | 56 |  |  |  |  |  |  |  |  |
| Fonkoua (39) | 2019 | 42 | F | Dental infection | *Cytomegalovirus* | Face, ears, upper arms, toes on both feet | Bilateral arms |  | 0 | 0 |  |  |  |  | 56 |  |  |  |
| Gast (40) | 2006 | 34 | F | Septic shock | *Haemophilus influenzae* | Bilateral fingers, buttocks, legs, feet |  |  | Bilateral BKA, autoamputation of finger tips | 66 |  | Split-thickness graft |  |  | 50 | Torulopsis glabrata sepsis, Serratia marcesens catheter tip infection |  |  |
| Gaucher (41) | 2010 | 32 | F | Septic shock(asplenic) | *Streptococccus pneumoniae* | Bilateral upper and lower limbs | Full-thickness | 19 | Bilateral BKA, bilateral below elbow | 118 |  | Cryopreserved human skin allografts from deceased donors, autografts | 19 |  |  |  | Yes | 69 |
| Gaucher (41) | 2010 | 20 | M | Septic shock | *Neisseria meningitidis* | Bilateral upper and lower limbs | Full-thickness | 23 | Bilateral BKA, partial finger | 61 |  | Cryopreserved human skin allografts from deceased donors, autografts | 23 |  | 130 |  |  |  |
| Gaucher (41) | 2010 | 19 | M | Septic shock | *Neisseria meningitidis* | Bilateral upper and lower limbs | Full-thickness | 13 | Bilateral BKA, partial finger | 61 |  | Cryopreserved human skin allografts from deceased donors, autografts | 13 |  | 216 |  |  |  |
| Ghosh (42) | 2010 | 34 | F | Septic shock(UTI) | *Escherichia coli* | Bilateral upper and lower limbs |  |  | Autoamputation of fingers/toes | 56 |  |  |  |  |  |  |  |  |
| Ghosh (42) | 2010 | 56 | M | Septic shock(pneumonia) | *Klebsiella pneumoniae* | Bilateral upper and lower limbs |  |  | Autoamputation of multiple fingers/toes | 56 |  |  |  |  |  |  |  |  |
| Ghosh (42) | 2010 | 75 | M | Septic shock | *Staphylococcus aureus* | Bilateral upper and lower limbs, ear, tip of nose |  |  | Autoamputation of fingers/toes | 56 |  |  |  |  |  |  |  |  |
| Ghosh (42) | 2010 | 50 | M | Septic shock(cholecystectomy) | Unknown | Bilateral upper and lower limbs |  |  | Bilateral lower limbs, fingers/toes | 60 |  |  |  |  |  |  |  |  |
| Ghosh (42) | 2010 | 53 | F | Septic shock(gastroenteritis) | *Escherichia coli* | Bilateral upper and lower limbs |  |  | Autoamputation of fingers/toes | 56 |  |  |  |  |  |  |  |  |
| Ghosh (42) | 2010 | 15 | F | Septic shock(UTI) | *Pseudomonas aeruginosa* | Bilateral upper and lower limbs, tip of nose | Full-thickness |  |  | 0 |  | Split-thickness graft |  |  |  |  |  |  |
| Hage-Sleiman (43) | 2019 | 21 | M | Meningitis(not vaccinated) | *Neisseria meningitidis*, C | Left index finger, bilateral big toes, left ear lobe, foreskin of penis | Circumcision of foreskin | 12 |  |  |  |  |  |  | 22 |  |  |  |
| Hagiya (44) | 2013 | 69 | M | Septic shock | *Streptococccus pneumoniae*, serotype 22 | Bilateral upper and lower limbs, face |  |  | Bilateral upper and lower limbs | 114 | 7 |  |  |  |  | Varicella reactivation |  |  |
| Har-El (45) | 1990 | 34 | M | Septic shock(pneumonia) | *Streptococccus pneumoniae* | Bilateral lower limbs, right nostril, upper and lower lips | Nose, lips | 13 | Bilateral AKA | 60 | 19 | Local flap |  |  |  | Right alar stenosis |  |  |
| Hassan (46) | 2008 | 71 | M | UTI | Unknown |  | Full-thickness |  |  | 0 |  |  |  |  |  |  |  |  |
| Hassan (46) | 2008 | 40 | F | Septic shock(asplenic) | Unknown |  |  |  | Bilateral BKA, bilateral forearm | 114 | 3 |  |  |  | 78 |  |  |  |
| Hassan (46) | 2008 | 21 | F | Septic shock | *Neisseria meningitidis* | Bilateral upper and lower limbs, buttocks |  |  | Right toes 3, 4, 5 and left toes 4, 5 metatarsal, bilateral BKA | 56 |  |  |  |  |  |  |  |  |
| Hassan (46) | 2008 | 62 | M | Septic shock | Unknown | Bilateral upper and lower limbs, trunk | Yes |  | 0 | 0 |  |  |  |  | 11 |  |  |  |
| Hassan (46) | 2008 | 17 | M |  | *Neisseria meningitidis* | Head to toe | Multiple full-thickness |  | 0 | 0 |  | Allograft, autograft |  |  | 20 |  |  |  |
| Hautekeete (47) | 1986 | 22 | F | Septic shock(asplenic) | *Streptococccus pneumoniae* | Nose, fingers, toes |  |  | Autoamputation, toe and finger | 6 |  |  |  |  | 42 |  |  |  |
| Hautekeete (47) | 1986 | 70 | F | Septic shock(asplenic) | *Streptococccus pneumoniae* | Bilateral upper and lower limbs, nose |  |  | 3 toes | 9 | 16 |  |  |  | 42 |  |  |  |
| Herzog (48) | 2010 | 48 | F | Meningitis(complement deficiency) | *Neisseria meningitidis* | Bilateral upper and lower limbs, fingers, toes, trunk, breasts, buttocks | Full-thickness | 23 | Bilateral transmetatarsals | 38 | 16 | 9 skin grafts |  | Right lower limb, chest, abdomen, back, buttock | 25 | Clostridium difficile, right eye thrombosis |  |  |
| Hogarth (49) | 2017 | 60 | M | Pre-syncope, malaise |  | right hip, glans penis, distal penile shaft, left hemiscrotum, bilateral medial thighs, suprapubic area | Partial glansectomy with debridment to Bucks fascia and sparing of the corporal bodies |  | 0 | 0 |  | Full-thickness graft |  | Distal penile shaft |  |  |  |  |
| Huemer (50) | 2004 | 26 | M | Liver failure(Wilson’s disease) | *Escherichia coli* | Bilateral lower limbs | Yes | 14 |  |  |  | Split-thickness graft | 28 |  | 57 |  |  |  |
| Ichimiya (51) | 2007 | 27 | F | Abortion | *Escherichia coli* | Back, buttock, hands, feet, | Back, buttock, gluteal and latissimus dorsi muscles |  | Both feet, right hand fingers 2,3,4,5 | 64 |  | Posterolateral thigh V-Y flap, split-thickness graft |  | Back, right hand, feet | 85 |  |  |  |
| Jackson (52) | 1998 | 42 | M | Septic shock(Factor V Leiden, asplenic) | *Streptococccus pneumoniae* | Fingers, toes, forehead, ears, nose |  |  | All toes, distal fingers | 38 |  |  |  |  |  |  |  |  |
| Jackson (52) | 1998 | 40 | F | Septic shock(Factor V Leiden, rectal fistula) | *Bacteroides fragilis, Fusobacterium species* | Fingers and toes |  |  | Toes | 28 |  |  |  |  |  |  |  |  |
| Jakob (53) | 2009 | 79 | F | URI | *Chlamydia pneumoniae* | Face, breasts, upper arms, knees, lower legs, back | Yes |  | 0 | 0 |  | Split-thickness graft | 16 | Left leg | 28 |  |  |  |
| Jha (54) | 2016 | 60 | F | Septic shock(COPD exacerbation) | *Streptococccus pneumoniae* | Bilateral lower limbs and forearms | Yes | 42 | Bilateral AKA, right hand | 87 | 42 |  |  |  |  |  |  |  |
| Jones (55) | 2012 | 15 | F | Septic shock |  | Left limb | Partial of left tibial compartment |  | 0 | 0 |  |  |  |  |  |  |  |  |
| Kahn (56) | 2011 | 16 | M | Macrophage activating syndrome |  | Right foot | Down to bone |  | 0 | 0 |  | ADM, split-thickness graft | 52 |  |  |  |  |  |
| Kato (57) | 2007 | 67 | F | Septic shock(malaria) | *Plasmodium falciparum* | All toes, left foot |  |  | Toes | 28 |  | Split-thickness graft |  |  | 56 |  |  |  |
| Kim (58) | 2007 | 46 | M | Septic shock | *Trichosporon asahii* | Left leg, left forearm, toe | Escharotomy | 13 | 0 | 0 |  | Split-thickness graft | 39 | Left lower leg, left forearm | 44 |  |  |  |
| Komatsu (59) | 2017 | 40 | M | Septic shock(not vaccinated, asplenic) | *Streptococccus pneumoniae* | Bilateral upper and lower limbs, head |  |  | Bilateral fingers, bilateral BKA | 84 |  |  |  |  | 292 |  |  |  |
| Kopinski (60) | 2014 | 57 | M | Septic shock | *Neisseria meningitidis* | Bilateral hands, both knees, left elbow, right ankle | Bilateral hands, both knees, left elbow, right ankle |  | 0 | 0 |  | 6 flaps, free ALT, free medial gastrocnemius, tendon reconstruction |  |  |  |  |  |  |
| Kuwahara (61) | 2017 | 60 | M | Septic shock(spleen hypoplasia) | *Streptococccus pneumoniae* | Bilateral upper and lower limbs, face, toes, 4 fingers, upper and lower lips, left side of nasal root | Lips |  | Toes, 4 fingers distal to the PIP joint, upper and lower lips | 32 | 40 | Orbicularis oris muscle-skin-mucosal pedicled flap, bipedicled flap |  | Upper and lower lips |  |  |  |  |
| Lyon (63) | 2011 | 67 | F | Septic shock(CKD) | Unknown | Left outer hip, both inner thighs | Larva therapy, followed by surgical of remaining necrotic tissue |  | 0 | 0 |  |  |  |  |  |  | Yes | 84 |
| MacLennan (64) | 2001 | 35 |  |  |  |  | Yes |  |  |  |  | Lateral arm flap |  | Right hand |  |  |  |  |
| MacLennan (64) | 2001 | 50 |  |  |  |  | Yes |  |  |  |  | Latissimus dorsi flap |  | Left lower limb exposed tibia |  |  |  |  |
| MacLennan (64) | 2001 | 19 |  |  |  |  | Yes |  |  |  |  | Latissiums dorsi, gracilis muscle flap |  | Left lower limb open tibia fracture, right lower limb exposed tibia |  |  |  |  |
| Michel (65) | 2013 | 60 | M | Septic arthritis, homeless, HIV+ | *Neisseria meningitidis* | Left leg, right foot | Knee washout, multiple I&D of lower limbs |  | 0 | 0 |  | Skin graft |  | Left lower limb | 62 | Wound infections, DVT, hospital acquired UTI |  |  |
| Moritz (66) | 2017 | 57 | M | Septic shock(autosplenectomy) | *Streptococccus pneumoniae* | Bilateral upper and lower limbs |  |  | Bilateral BKA, 3 fingers | 65 | 14 |  |  |  | 365 |  |  |  |
| Morris (1) | 2013 | 30 | F | Septic shock(tooth abscess) | *Neisseria meningitidis* | Bilateral upper and lower limbs, right hand | 4 compartment fasciotomy of left lower limb | <1 | Bilateral BKA, fingers 1 and 2 of right hand | 61 |  |  |  |  |  |  |  |  |
| Nolan (67) | 2001 | 18 | F | Chickenpox | *Varicella* | Thighs, calves, upper limbs, flanks, hands |  |  | Several digits | 14 | 28 |  |  |  |  | PE |  |  |
| Note (68) | 2018 | 60 | F | Septic shock | *Streptococccus pyogenes* | Bilateral lower limbs | Fasciotomy bilateral lower limbs | 1 | 0 | 0 |  | Autografts | 42 | Bilateral lower limbs | 42 |  |  |  |
| Okamura (69) | 2016 | 22 | F | Medication-related | Unknown | Right leg, torso | Fasiotomy, removal of necrotic tissue | 4 | 0 | 0 |  | Skin graft | 70 |  |  |  |  |  |
| Ozmen (70) | 2005 | 68 | F | Breast necrosis | Unknown | Left breast, right lower limb | Daily breast excision |  | 0 | 0 |  |  |  |  | 38 | Right lower limb DVTs | Yes |  |
| Pino (6) | 2016 | 86 | M | Sepsis(cholangitis) | *Escherichia coli, Enterococcus gallinarum* | Bilateral upper and lower limbs | Yes | 28 | Autoamputation of multiple fingers, revision of left index finger, middle finger, right thumb, middle finger, ring finger | 14 | 28 |  |  |  | 56 | Wound infections |  |  |
| Pollard (71) | 2008 | 19 | F | Septic shock | *Neisseria meningitidis* | Bilateral lower limbs | Bilateral legs down to tibia removing periosteum | 5 | Right BKA | 56 |  | ADM, split-thickness grafts, tissue flaps | 5, 32, 32-84 | Bilateral ankles | 224 | osteomyelitis of right ankle, BKA |  |  |
| Redett (72) | 2000 | 19 | M | Septic shock(URI) | *Neisseria meningitidis*, serogroup W135 | Face, back, buttocks, abdomen, legs, elbows | Infected eschars of both elbows | 21 | 0 | 0 |  | Bilateral latissimus dorsi | 28 | Bilateral elbows | 46 | Bilateral elbow infections, left elbow contracture |  |  |
| Rintala (73) | 2000 | 20 | F |  | *Neisseria meningitidis* | Both legs, tips of right hand fingers |  |  |  |  |  |  |  |  |  |  |  |  |
| Rintala (73) | 2000 | 26 | F |  | *Capnocytophaga canimorsus* | 2 fingers, all toes |  |  |  |  |  |  |  |  |  |  |  |  |
| Rintala (73) | 2000 | 53 | F |  | Unknown | Several fingers and toes |  |  |  |  |  |  |  |  |  |  |  |  |
| Roughton (8) | 2011 | 22 | F |  | *Neisseria meningitidis* |  | 2 fasciotomies | <1 | 2 limbs | 60 |  |  |  |  |  |  |  |  |
| Roughton (8) | 2011 | 32 | F |  | *Plasmodium falciparum* |  |  |  | Bilateral upper and lower limbs | 114 |  |  |  |  |  |  |  |  |
| Roughton (8) | 2011 | 30 | F |  | *Neisseria meningitidis* |  |  |  | 2 limbs | 60 |  |  |  |  |  |  |  |  |
| Roughton (8) | 2011 | 38 | M |  | *Staphylococcus aureus* |  | Fasciotomy | <1 | 0 | 0 |  |  |  |  |  |  |  |  |
| Roughton (8) | 2011 | 41 | F |  | *Neisseria meningitidis* |  |  |  | 2 limbs | 60 |  |  |  |  |  |  |  |  |
| Roughton (8) | 2011 | 40 | F |  | *Streptococccus pneumoniae* |  |  |  | Bilateral upper and lower limbs | 114 |  |  |  |  |  | Sepsis | Yes | 84 |
| Roughton (8) | 2011 | 21 | F |  | *Neisseria meningitidis* |  | Fasciotomy | <1 |  | 0 |  |  |  |  |  |  | Yes |  |
| Roughton (8) | 2011 | 29 | M |  | *Streptococccus pneumoniae* |  | Fasciotomy | <1 |  | 0 |  |  |  |  |  |  | Yes |  |
| Roughton (8) | 2011 | 16 | M |  | *Streptococccus pneumoniae* |  | Fasciotomy | <1 |  | 0 |  |  |  |  |  |  | Yes |  |
| Saraceni (74) | 2013 | 46 | F | Septic shock(pneumonia, asplenic) | *Streptococccus pneumoniae* | Bilateral upper and lower limbs, trunk, chest face, neck | Multiple areas |  | Bilateral BKA, left hand, partial of digits 2 and 3 at the distal interphalangeal joint of the right hand | 85 |  | Multiple skin grafts |  |  | 48 |  |  |  |
| Shah (75) | 2016 | 42 | M | Septic shock, encephalitis | West Nile Virus |  |  |  | Bilateral upper and lower limbs | 114 |  |  |  |  |  | Enterococcus faecalis, Trichosporon spp, Candida albicans, Bacteroides fragilis infections | Yes |  |
| Shapiro (76) | 2009 | 40 | F | Septic shock | *Staphylococcus aureus* |  | Webplasty of both hands, excision of heterotopic bone |  | Bilateral BKA, bilateral metacarpophalangeal | 114 |  | Multiple skin grafts |  |  | 197 | Nosocomial infections, sacral pressure ulcer, heterotopic ossification |  |  |
| Shapiro (76) | 2009 | 43 | F | Septic shock, postpartum | *Streptococccus pyogenes* |  | Fasciotomies of right upper limb and bilateral lower limb |  | Bilateral AKA, right transhumeral | 94 |  | Multiple skin grafts |  |  | 88 |  |  |  |
| Shapiro (76) | 2009 | 19 | F | Meningitis | *Neisseria meningitidis* |  |  |  | Bilateral BKA, bilateral transradial | 90 |  |  |  |  | 75 |  |  |  |
| Shapiro (76) | 2009 | 24 | F | Septic shock, postpartum | *Streptococccus pyogenes* |  |  |  | Bilateral AKA, right transradial, left transhumeral | 119 |  |  |  |  | 99 | Pelvic abscess requiring total abdominal hysterectomy and bilateral salpingo-oophorectomies, DVT |  |  |
| Singer (77) | 1990 | 62 | F | Sepsis | *Streptococccus pneumoniae* |  | Radical of lower limbs |  | Bilateral fingers of both hands, bilateral AKA | 88 |  |  |  |  |  | Left leg abscess | Yes |  |
| Smith (78) | 1997 | 16 | F | Septic shock | *Neisseria meningitidis*, C |  | Yes |  | 0 | 0 |  | Multiple skin grafts |  |  | 56 |  |  |  |
| Smith (78) | 1997 | 19 | M | Septic shock | *Neisseria meningitidis*, B |  | Fasciotomies in bilateral legs and right arm |  | Bilateral lower limbs | 56 |  |  |  |  | 112 |  |  |  |
| Srinivasan (79) | 2016 | 68 | F | Septic shock | *Streptococccus pneumoniae* |  |  |  | Left forearm, right arm below elbow, left AKA | 88 |  |  |  |  |  | Facial deformity |  |  |
| Talwar (80) | 2012 | 30 | M | Sepsis | *Leptospirosis* |  | Minimal |  | 1 finger | 3 |  |  |  |  |  |  |  |  |
| Tanosaki (81) | 2014 | 72 | M | Septic shock, meningitis | *Neisseria meningitidis* |  | Multiple |  | Partial bilateral fingers, bilateral BKA | 64 |  |  |  |  | 55 |  |  |  |
| Urushidate (82) | 2012 | 48 | M | Septic shock(neurosurgery) | *Klebsiella pneumoniae* |  |  |  | 0 | 0 |  | Bilateral orbicularis oris myocutaneous flap, free forearm flap, forehead flap | 224 |  |  |  |  |  |
| Van De Yen (85) | 2004 | 55 | F | Septic shock(dog bite) | *Capnocytophaga canimorsus* |  |  |  | Bilateral hands, left forefoot, right BKA | 84 | 28 |  |  |  |  |  |  |  |
| Van Der Horst (83) | 1968 | 18 | F | Abortion(self-performed) | *Escherichia coli* |  |  |  | Bilateral BKA | 56 |  | Skin graft |  |  |  |  |  |  |
| Yamagishi (84) | 2018 | 44 | M | Septic shock | *Gemella bergeri* |  |  |  | Bilateral BKA | 56 |  |  |  |  | 121 | Infections |  |  |
| Yoshimoto (86) | 2013 | 52 | M | Septic shock | *Streptococccus pneumoniae* |  | Nose, other facial areas |  | Bilateral transradial, left BKA, right AKA | 116 |  |  |  |  | 56 |  |  |  |
| Yoshimoto (86) | 2013 | 62 | M | Septic shock | *Streptococccus pneumoniae* |  | Upper lip |  | Bilateral transradial, bilateral AKA | 118 |  |  |  |  | 84 | Shoulder joint contractures |  |  |
| Zerbib (87) | 2007 | 63 | M | Septic shock(not vaccinated, asplenic) | *Streptococccus pneumoniae* |  |  |  | Partial of feet and fingers | 56 | 56 | Skin graft |  |  |  |  |  |  |

LOS: length of stay, BKA: below-knee amputation, AKA: above knee amputation, ADM: acellular dermal matrix, UTI: urinary tract infection, URI: upper respiratory infection, PE: pulmonary embolism, DVT: deep vein thrombosis, CKD: chronic kidney disease, ALT: anterolateral thigh, HIV: human immunodeficiency virus
