# Supplementary material for: Surgical outcomes in adults with purpura fulminans: a systematic review and patient-level meta-synthesis
Source: Burns Trauma. 2019 Oct 18;7:30. doi: 10.1186/s41038-019-0168-x (PMC6798408; doi:10.1186/s41038-019-0168-x)
Supplement: Supplementary file 3 — : Table S2. Cohort studies with surgical outcomes of purpura fulminans (DOCX 50 kb) [file 41038_2019_168_MOESM3_ESM.docx]

Supplemental Table 2. Cohort studies with surgical outcomes of purpura fulminans

| Author | Year | Sample size | Study design | Age  Mean±SD | Sex | Etiology | Infectious organism | Necrosis | Amputation  Mean±SD | Mortality | Time to mortality (days)  Mean±SD |
| --- | --- | --- | --- | --- | --- | --- | --- | --- | --- | --- | --- |
| Contou (28) | 2018 | 51 | Retrospective cohort | 39±34 | Males=29  Females=22 | Asplenic=12 | *Neisseria meningitidis*(n=23)  *Streptococccus pneumoniae* (n=25)  Other(n=3) |  | 3±3 limbs | 5 |  |
| Lerolle (62) | 2013 | 20 | Prospective cohort | 68±23 | Males=12  Female=8 | Septic shock, site of infection  Lungs=7  Abdominal=4  Meningitis=2  Other=7 | *Neisseria meningitidis*(n=2),  *Streptococccus pneumonia*(n=4)  Other *Streptococcus*(n=5)  *Staphylococcus aureus*(n=2)  Gram-negative bacilli(n=3)  Other(n=5) | Limbs bilaterally=17  Trunk=9  Superior limbs=11  Head=3 | 2 patients with multiple limb | 16 | 2±2 |

SD: standard deviation
