# Supplementary material for: Surgical outcomes in adults with purpura fulminans: a systematic review and patient-level meta-synthesis
Source: Burns Trauma. 2019 Oct 18;7:30. doi: 10.1186/s41038-019-0168-x (PMC6798408; doi:10.1186/s41038-019-0168-x)
Supplement: Supplementary file 4 — : Table S3. Methodological quality and synthesis of case series and case reports system [10] (DOCX 131 kb) [file 41038_2019_168_MOESM4_ESM.docx]

Supplemental Table 3. Methodological Quality and Synthesis of Case Series and Case Reports System(10)

| Author | Selection | Ascertainment | | Causality | Reporting |
| --- | --- | --- | --- | --- | --- |
|  | Does the patient(s) represent(s) the whole experience of the investigator (centre) or is the selection method unclear to the extent  that other patients with similar presentation may not have been reported? | Was the exposure adequately ascertained? | Was the outcome adequately ascertained? | Was follow-up long enough for outcomes to occur? | Is the case(s) described with sufficient details to allow other investigators to replicate the research or to allow practitioners make  inferences related to their own practice? |
| Agarwal (12) | Yes | Yes | Yes | No | No |
| Ahmad (13) | Yes | Yes | Yes | No | Yes |
| Amara (15) | Yes | Yes | Yes | No | No |
| Andreasen (14) | Yes | Yes | Yes | No | Yes |
| Arevalo (16) | Yes | Yes | Yes | No | Yes |
| Arnaiz-Garcia (17) | Yes | Yes | Yes | Yes | No |
| Bendapudi (18) | Yes | Yes | Yes | No | No |
| Bhatti (19) | Yes | No | Yes | Yes | Yes |
| Bischof (20) | Yes | Yes | Yes | Yes | No |
| Bollero (21) | Yes | Yes | Yes | Yes | Yes |
| Borges (22) | Yes | Yes | Yes | No | Yes |
| Chasan (23) | Yes | Yes | Yes | No | Yes |
| Choi (24) | Yes | Yes | Yes | No | Yes |
| Christiansen (25) | Yes | Yes | Yes | Yes | Yes |
| Chu (26) | Yes | Yes | Yes | No | No |
| Cone (27) | Yes | Yes | Yes | Yes | No |
| Dautzenberg (29) | Yes | Yes | Yes | Yes | Yes |
| Davis (30) | Yes | Yes | Yes | Yes | Yes |
| De Salvia (31) | Yes | Yes | Yes | Yes | No |
| Dedy (32) | Yes | Yes | Yes | Yes | Yes |
| Desai (33) | Yes | Yes | Yes | Yes | No |
| Duteille (34) | Yes | No | Yes | Yes | Yes |
| Dykstra (35) | Yes | Yes | Yes | Yes | Yes |
| El-Agwany (36) | Yes | Yes | Yes | Yes | Yes |
| Endo (37) | Yes | Yes | Yes | Yes | Yes |
| Eng (38) | Yes | Yes | Yes | No | Yes |
| Fonkoua (39) | Yes | Yes | Yes | No | Yes |
| Gast (40) | Yes | Yes | Yes | No | Yes |
| Gaucher (41) | Yes | Yes | Yes | No | Yes |
| Ghosh (42) | Yes | Yes | Yes | No | Yes |
| Hage-Sleiman (43) | Yes | Yes | Yes | No | Yes |
| Hagiya (44) | Yes | Yes | Yes | No | Yes |
| Har-El (45) | Yes | Yes | Yes | No | Yes |
| Hassan (46) | Yes | Yes | Yes | No | Yes |
| Hautekeete (47) | Yes | Yes | Yes | No | Yes |
| Herzog (48) | Yes | Yes | Yes | No | Yes |
| Hogarth (49) | Yes | No | Yes | Yes | Yes |
| Huemer (50) | Yes | Yes | Yes | No | Yes |
| Ichimiya (51) | Yes | Yes | Yes | No | Yes |
| Jackson (52) | Yes | Yes | Yes | No | No |
| Jakob (53) | Yes | Yes | Yes | No | Yes |
| Jha (54) | Yes | Yes | Yes | No | No |
| Jones (55) | Yes | No | Yes | No | No |
| Kahn (56) | No | No | Yes | No | Yes |
| Kato (57) | Yes | Yes | Yes | No | Yes |
| Kim (58) | Yes | Yes | Yes | No | Yes |
| Komatsu (59) | Yes | Yes | Yes | No | No |
| Kopinski (60) | Yes | Yes | Yes | No | Yes |
| Kuwahara (61) | Yes | Yes | Yes | Yes | Yes |
| Lyon (63) | Yes | No | Yes | Yes | Yes |
| MacLennan (64) | No | No | Yes | Yes | Yes |
| Michel (65) | Yes | Yes | Yes | No | Yes |
| Moritz (66) | Yes | Yes | Yes | No | Yes |
| Morris (1) | Yes | Yes | Yes | No | Yes |
| Nolan (67) | Yes | Yes | Yes | No | Yes |
| Note (68) | Yes | Yes | Yes | No | Yes |
| Okamura (69) | Yes | No | Yes | Yes | Yes |
| Ozmen (70) | Yes | No | Yes | Yes | No |
| Pino (6) | Yes | Yes | Yes | No | Yes |
| Pollard (71) | Yes | Yes | Yes | No | Yes |
| Redett (72) | Yes | Yes | Yes | Yes | Yes |
| Rintala (73) | Yes | Yes | Yes | Yes | Yes |
| Roughton (8) | Yes | Yes | Yes | No | No |
| Saraceni (74) | Yes | Yes | Yes | No | No |
| Shah (75) | Yes | Yes | Yes | No | No |
| Shapiro (76) | Yes | Yes | Yes | No | Yes |
| Singer (77) | Yes | Yes | Yes | No | Yes |
| Smith (78) | Yes | Yes | Yes | No | Yes |
| Srinivasan (79) | Yes | Yes | Yes | Yes | No |
| Talwar (80) | Yes | Yes | Yes | Yes | No |
| Tanosaki (81) | Yes | Yes | Yes | Yes | No |
| Urushidate (82) | Yes | Yes | Yes | Yes | Yes |
| Van De Yen (85) | Yes | Yes | Yes | No | Yes |
| Van Der Horst (83) | Yes | Yes | Yes | No | No |
| Yamagishi (84) | Yes | Yes | Yes | No | Yes |
| Yoshimoto (86) | Yes | Yes | Yes | No | Yes |
| Zerbib (87) | Yes | Yes | Yes | No | No |
